# Ecophysiological and Transcriptomic Responses of Mangrove Seedlings to Environmentally Relevant Cu–Pb–Cd Combined-Metal Stress

**DOI:** 10.3390/plants15203197

**Published:** 2026-10-18

**Authors:** Yueyue Zhou, Guiwu Huang, Yaling Zhao, Yan Yu

**Affiliations:** 1 Department of Environment Engineering, College of Chemistry and Chemical Engineering, Zhoukou Normal University, Zhoukou 466001, China; 2 School of Computer Science and Technology, Zhoukou Normal University, Zhoukou 466001, China; 15138221250@163.com; 3 College of Chemistry and Chemical Engineering, Zhoukou Normal University, Zhoukou 466001, China

**Keywords:** mangrove wetland, Cu–Pb–Cd combined-metal stress, plant abiotic stress, plant metal uptake, chlorophyll fluorescence, transcriptomic response, phytoremediation

## Abstract

Mangrove wetlands are important coastal blue-carbon ecosystems that are increasingly exposed to combined-metal stress by copper (Cu), lead (Pb), and cadmium (Cd). However, how mangrove seedlings coordinate metal exclusion, redox regulation, and photosynthetic adjustment under environmentally relevant metal mixtures remains insufficiently resolved. In this study, five dominant mangrove species, *Bruguiera gymnorrhiza*, *Rhizophora stylosa*, *Kandelia obovata*, *Aegiceras corniculatum*, and *Avicennia marina*, were exposed to a gradient of Cu–Pb–Cd combined stress under hydroponic and soil-culture systems for 30 days. We measured tissue metal accumulation, growth, leaf hydrogen peroxide (H_2_O_2_), chlorophyll content and fluorescence, and examined root and leaf transcriptomic patterns. Roots generally accumulated more Cu, Pb, and Cd than leaves, while seedlings grown in soil accumulated less metal than those in solution. At higher mixture concentrations, *A. corniculatum* and *K. obovata* showed greater visible injury, H_2_O_2_ accumulation, and reductions in chlorophyll and PSII-related fluorescence parameters. *B. gymnorrhiza* and *A. marina* showed comparatively smaller changes. Root- and leaf-associated transcriptomic patterns suggested possible involvement of transport-related and photosynthesis-related pathways, although these associations do not establish causal mechanisms. These findings indicate that mangrove tolerance to Cu–Pb–Cd combined-metal stress is jointly regulated by root metal exclusion, H_2_O_2_-mediated redox changes, and photoprotective adjustment, thereby clarifying plant-level mechanisms of abiotic stress tolerance under combined metal exposure. The results identify *B. gymnorrhiza* and *A. marina* as preliminary candidates for further evaluation under field conditions.

## 1. Introduction

Mangrove forests are unique intertidal wetland ecosystems distributed in tropical and subtropical coastal regions [1]. They provide irreplaceable ecological services including coastal protection (windbreak, wave attenuation, tsunami resistance), sediment accretion promotion, biodiversity conservation, and coastal ecological security maintenance [2]. As a major coastal “blue carbon” ecosystem, mangroves contribute approximately 5% of global coastal carbon fixation despite covering only 0.7% of global coastal areas, with a carbon sequestration capacity 2–10 times higher than that of terrestrial forests, making them critical for mitigating global climate change [3]. Photosynthesis, the fundamental process of plant biomass production and carbon sequestration, directly determines mangrove primary productivity and carbon sequestration efficiency, thus becoming a core research focus in mangrove ecology and environmental science.

Early research on mangrove photosynthesis examined canopy gas exchange, environmental constraints, and plant productivity. Subsequent model-based studies and field observations revealed strong correlations between mangrove photosynthetic efficiency and environmental factors such as light intensity, temperature, salinity, and tidal fluctuations, with salinity identified as a key abiotic stressor inducing osmotic stress and ion toxicity [4]. Since its discovery, photosynthesis has been a pivotal topic in plant physiology [5]. Most previous mangrove photosynthesis studies concentrated on the effects of salt stress and temperature, exploring adaptive mechanisms [6,7]. However, Cu, Pb, and Cd exposure has become an important combined abiotic stress in mangrove wetlands, posing risks to plant growth, photosynthetic stability, and ecosystem resilience [8,9].

Heavy metal pollution in mangrove wetlands has attracted growing attention due to its persistence, bioaccumulation, and toxicity [9,10,11]. Studies have shown that heavy metal contents in Chinese mangrove sediments follow the order Cu > Pb > Cd [12], indicating that mangroves in natural environments are frequently exposed to combined heavy metal contamination rather than single metal stress. Unlike essential heavy metals (e.g., Cu, which participates in cytochrome c oxidase and plastocyanin synthesis and is critical for photosynthetic electron transport), non-essential heavy metals (Cd, Pb) have no biological functions and are highly toxic even at low concentrations. These metals can interfere with nutrient uptake, photosynthetic metabolism, and antioxidant system function, ultimately inhibiting growth and inducing cell death [13,14].

One potential consequence of metal-related oxidative stress is membrane lipid peroxidation, which can compromise cell membrane integrity. Under metal stress, the net photosynthetic rate decreases due to disrupted photosynthetic metabolism balance and damaged electron transport systems [13,15]. A key plant response to heavy metals is the overproduction of reactive oxygen species (ROS) such as H_2_O_2_, superoxide anion (O_2_·^−^), and hydroxyl radical (OH), inducing oxidative stress when ROS generation exceeds scavenging capacity [16]. Heavy metals inhibit photosynthesis mainly by interfering with photosynthetic electron transport and undermining chlorophyll integrity: excess Cu binds to sulfhydryl groups of thylakoid membrane proteins, damaging the structure and blocking electron transfer between Photosystem I (PSI) and PSII [15]. Pb replaces Mg^2+^ in chlorophyll molecules, destroying light absorption capacity.

However, the molecular mechanisms by which Cu-Pb-Cd combined stress affects mangrove photosynthesis, especially through metal transport regulation and redox regulation, remain poorly understood in true mangrove species. Moreover, most previous studies adopted stress concentrations deviating from natural environmental levels, limiting the practical application of research results.

Published work proposes several possible routes by which metals may affect photosynthetic electron transport:

Altered functioning of the PSII oxygen-evolving complex and associated electron donation to PSII;

Disruption of chlorophyll–protein complexes, potentially affecting the distribution and transfer of absorbed light energy;

Changes in thylakoid-associated electron-transport components, which may alter photosynthetic electron flow [17].

Notably, most existing studies focus on single metal stress, while research on combined stress—more representative of natural pollution scenarios—is limited. Effects observed during mixture exposure cannot be classified as synergistic, additive, or antagonistic without corresponding single-metal or factorial controls. The relationship among metal accumulation, H_2_O_2_ levels, transcript abundance, and photosynthetic measurements remains a useful topic for further experiments [18].

To address these knowledge gaps and support mangrove conservation and ecological restoration in polluted coastal areas, this study selected five dominant true mangrove species widely distributed in southern China (*B. gymnorrhiza*, *R. stylosa*, *K. obovata*, *A. corniculatum*, *A. marina*) and subjected them to gradient Cu-Pb-Cd combined stress under hydroponic and soil cultivation conditions. Reported sediment concentrations from southern Chinese mangroves informed the selection of a laboratory exposure gradient; sediment values expressed as mg/kg were not treated as equivalent to dissolved concentrations expressed as mg/L.

Accordingly, this study examined the ecophysiological and transcriptomic responses of five dominant true mangrove species widely distributed in southern China (*B. gymnorrhiza*, *R. stylosa*, *K. obovata*, *A. corniculatum*, and *A. marina*) under gradient Cu–Pb–Cd combined stress. Hydroponic culture allowed controlled exposure to nominal concentrations of added dissolved salts, whereas the soil culture provided a contrasting growth system in which plant metal uptake and stress responses could be compared; dissolved and pore-water metal fractions were not measured.

The objectives were to (1) compare Cu, Pb, and Cd accumulation and translocation among species and cultivation systems; (2) evaluate growth, oxidative status, chlorophyll content, and chlorophyll fluorescence responses; (3) describe transcriptomic associations with transport- and photosynthesis-related processes; and (4) identify species showing comparatively smaller short-term responses as candidates for subsequent field evaluation.

## 2. Results

### 2.1. Cu, Pb, and Cd Accumulation in Roots and Leaves

**Figure 1:**
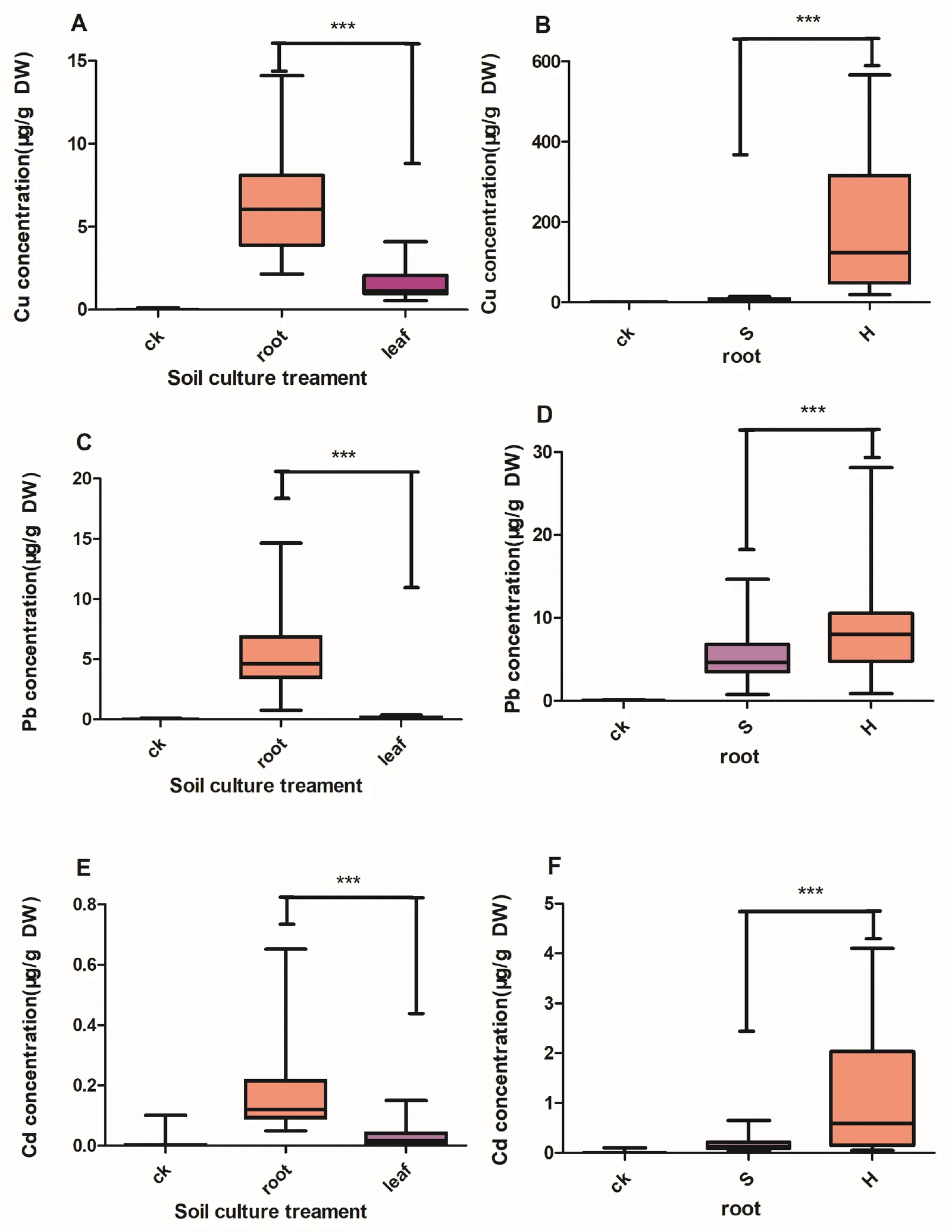
The Cu/Pb/Cd contents in the leaves and roots of five mangroves with two cultivation methods. (**A**) The Cu contents in the leaves and roots of five mangroves in Soil culture treatment. (**B**) The Cu contents in the roots of five mangroves with two cultivation methods. (**C**) The Pb contents in the leaves and roots of five mangroves in Soil culture treatment. (**D**) The Cu contents in the roots of five mangroves with two cultivation methods. (**E**) The Cd contents in the leaves and roots of five mangroves in Soil culture treatment. (**F**) The Cu contents in the roots of five mangroves with two cultivation methods. ck (control): Cu = 0, Pb = 0, Cd = 0 mg/L. S: Soil culture treatment. H: Hydroponic culture treatment. ***: There was a statistically significant difference between the data of the two groups.

Heavy metal accumulation in mangrove seedlings showed significant tissue specificity: root contents of Cu, Pb, and Cd were markedly higher than leaf contents in all species and treatments (*p* < 1 × 10^−4^, Table S1). Root metal content increased significantly with an increasing external concentration in both cultivation systems, showing a clear concentration-dependent relationship, while leaf accumulation varied only slightly (Figure 1, Figures S1–S3). Cultivation method significantly affected heavy metal uptake: hydroponically grown seedlings accumulated substantially more heavy metals in roots than soil-cultivated seedlings (Table S1). Specifically, root Cu content under hydroponic conditions was 36-fold higher, root Pb content 2-fold higher, and root Cd content 6-fold higher than under soil cultivation. Leaf Cu and Pb contents were also higher in hydroponic seedlings (*p* < 1 × 10^−4^), while leaf Cd content showed no significant difference between cultivation methods (*p* = 0.996).

Under hydroponic conditions, interspecific differences in root heavy metal accumulation were evident (Table S2):

Root Cu content order: *A. marina* ≈ *K. obovata* > *A. corniculatum* > *B. gymnorrhiza* > *R. stylosa*;

Root Pb content order: *K. obovata* > *A. corniculatum* > *R. stylosa* ≈ *A. marina* > *B. gymnorrhiza*;

Root Cd content order: *K. obovata* ≈ *R. stylosa* > *A. corniculatum* > *B. gymnorrhiza* > *A. marina*.

Dunn’s multiple comparison test revealed that *A. marina* had significantly lower root Cd content than *B. gymnorrhiza* (*p* < 0.001), *R. stylosa* (*p* < 0.05), and *K. obovata* (*p* < 1 × 10^−3^), consistent with lower measured root Cd accumulation under the specified treatments (Table S2). The translocation factor (TF = leaf heavy metal content/root heavy metal content) of all species was <0.1, suggesting limited root-to-leaf translocation during the 30-day experimental period.

### 2.2. Morphological Responses to Cu-Pb-Cd Stress

After 30 days of treatment, significant differences in seedling morphology were observed between the two cultivation methods. No mortality was recorded in soil-cultivated seedlings across all heavy metal concentrations, with negligible leaf damage and a 100% survival rate, showing less visible injury in soil-grown seedlings under the present laboratory conditions. In contrast, hydroponically grown seedlings showed severe morphological damage under high-concentration stress (C4): leaves of *A. corniculatum* and *K. obovata* turned yellow, wilted, and abscised, with soft and drooping stems. The order of damage severity under hydroponic conditions was *A. corniculatum* > *K. obovata* > *R. stylosa* > *A. marina* > *B. gymnorrhiza* (Figure S4).

Seedling height was not significantly affected by heavy metal stress in soil-cultivated seedlings (*p* > 0.05, Table S3), with only a 2.30–5.60% decrease under C4 treatment compared to the control. In hydroponic seedlings, height decreased significantly with an increasing heavy metal concentration (*p* < 0.05). Compared to the control, *A. corniculatum* and *K. obovata* height decreased by 59.2% and 35.8%, respectively, under C4 treatment, while *A. marina* and *B. gymnorrhiza* showed only 10.2% and 12.5% decreases, reflecting obvious interspecific tolerance differences.

### 2.3. Leaf H_2_O_2_ Content During Cu–Pb–Cd Mixture Exposure

**Figure 2:**
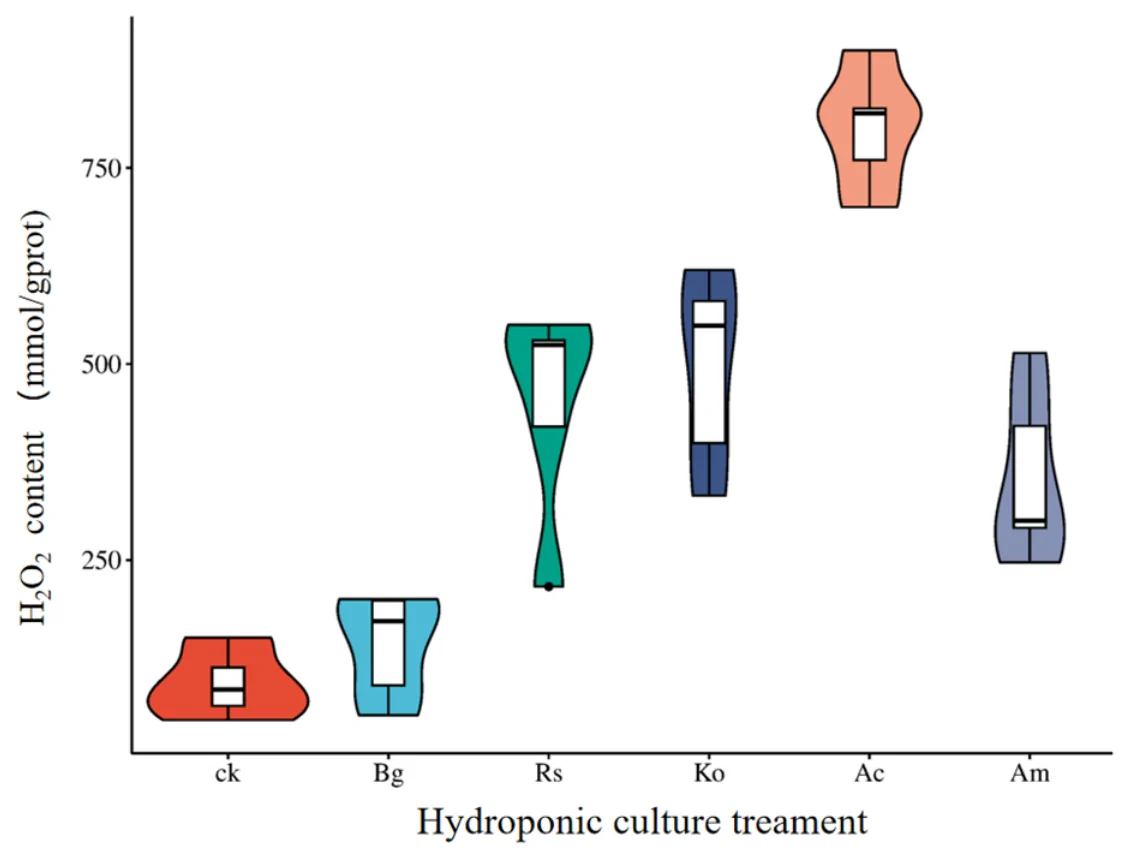
The H_2_O_2_ content of five mangroves with hydroponic culture treatment.

Leaf H_2_O_2_ content increased significantly with an increasing combined heavy metal concentration in hydroponic seedlings (Figure 2). At the highest concentration (C4), all species showed significantly higher H_2_O_2_ levels than the control (*p* < 0.05). The order of H_2_O_2_ content at C4 was *A. corniculatum* > *K. obovata* > *R. stylosa* > *A. marina* > *B. gymnorrhiza*, consistent with the severity of morphological damage. Higher H_2_O_2_ concentrations are consistent with increased oxidative burden, but do not establish that antioxidant enzymes were impaired. No significant differences in H_2_O_2_ content were observed between treatments in soil-cultivated seedlings, consistent with the weaker observed response in soil-grown seedlings (Figure S5).

### 2.4. Leaf Chlorophyll Content Responses to Stress

**Figure 3:**
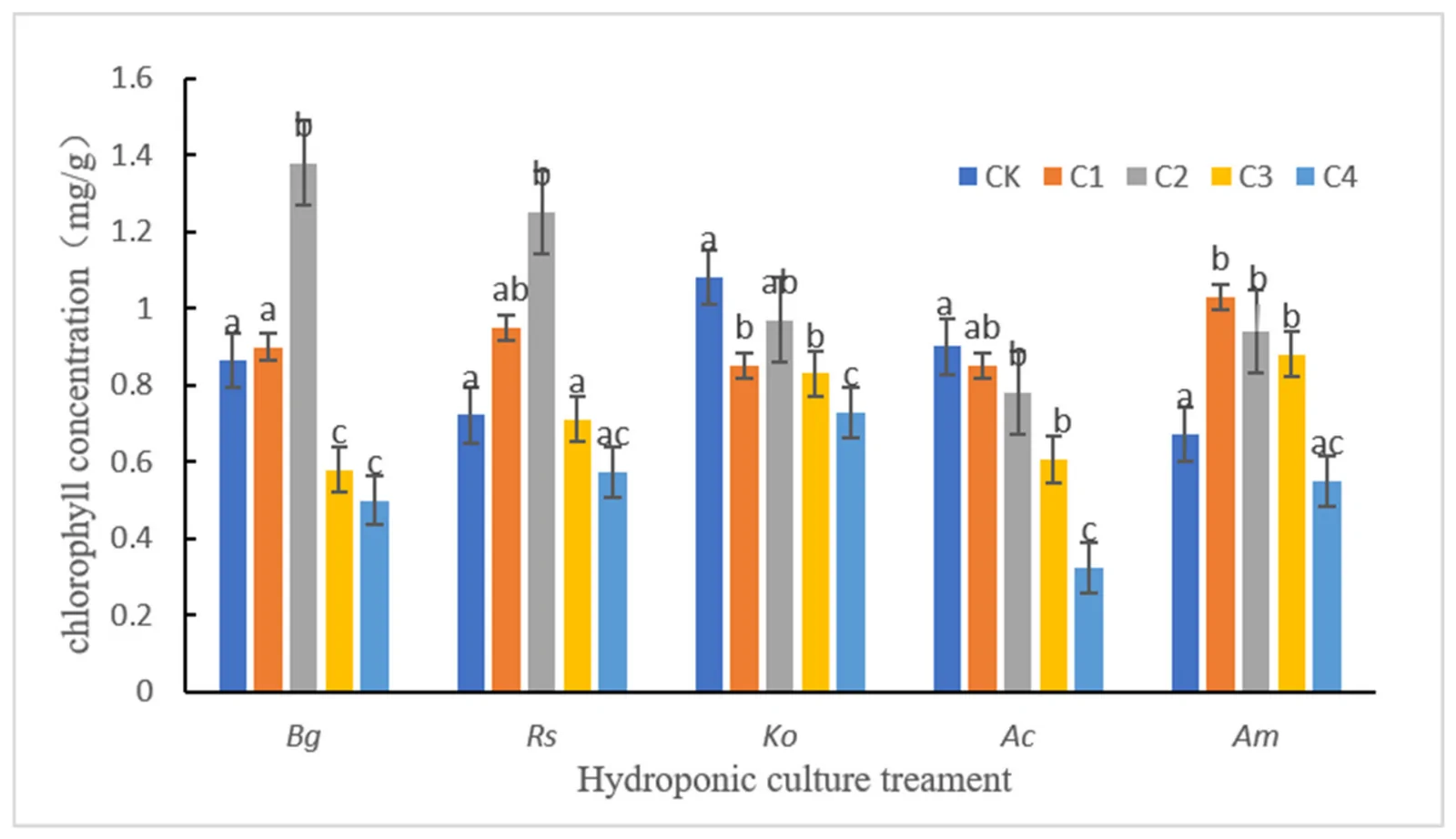
Chlorophyll concentration of five mangrove species under hydroponic combined-metal exposure. Values and error bars indicate mean ± standard deviation (*n* = 3). Different letters indicate significant differences among treatments within the same species (*p* < 0.05).

**Figure 4:**
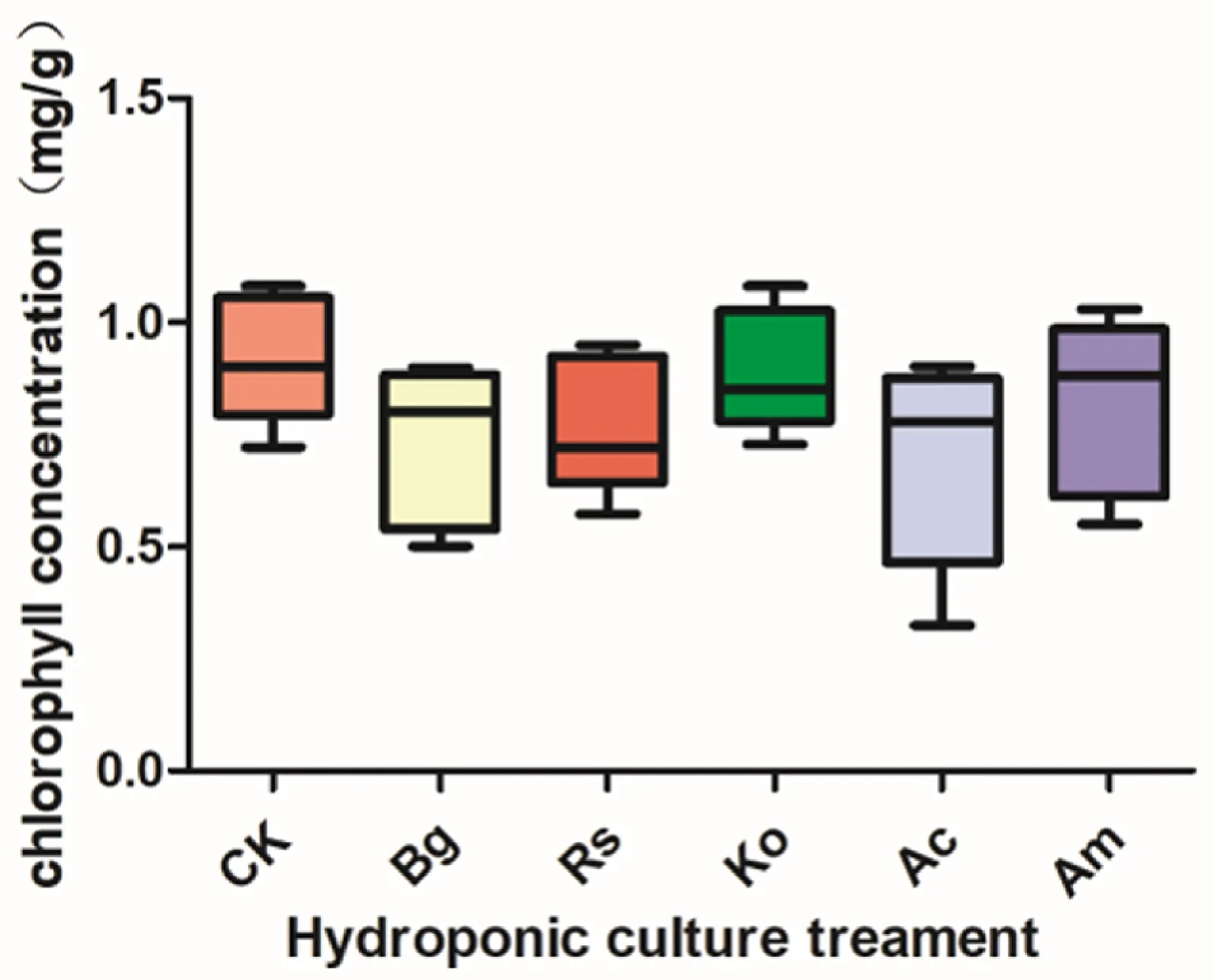
Chlorophyll concentration of five mangrove species under hydroponic combined-metal exposure.

Under hydroponic conditions, chlorophyll content showed a concentration-dependent response (Figure 3 and Figure 4): low concentration (C1) slightly increased chlorophyll content in tolerant species (*A. marina*, *B. gymnorrhiza*), a possible non-monotonic response that does not establish a specific physiological mechanism. High concentrations (C3–C4) significantly reduced the chlorophyll content (*p* < 0.05), although the rates of chlorophyll synthesis and degradation were not directly measured. Compared to the control, *A. corniculatum* and *K. obovata* chlorophyll content decreased by 63.9% and 32.7%, respectively, under C4 treatment, while *B. gymnorrhiza*, *R. stylosa*, and *A. marina* showed milder decreases (12.6%, 20.6%, 18.1%). No significant chlorophyll content changes were observed in soil-cultivated seedlings, consistent with minimal morphological damage and low oxidative stress.

### 2.5. Leaf Chlorophyll Fluorescence Responses

Combined heavy metal stress induced consistent changes in the chlorophyll fluorescence parameters of hydroponic seedlings (Figure S6), indicating changes in PSII photochemical performance:

F_v_/F_m_ (maximum quantum yield of PSII): Decreased significantly with an increasing heavy metal concentration, consistent with reduced maximum PSII photochemical efficiency; the precise site of any structural injury was not examined. Under C4 treatment, *A. corniculatum* and *K. obovata* F_v_/F_m_ decreased by 42.3% and 31.5%, respectively, while *A. marina* and *B. gymnorrhiza* showed only 15.2% and 18.7% decreases, confirming interspecific differences in PSII stability.

qP (photochemical quenching) and ETR (electron transport rate): Both parameters decreased significantly with an increasing concentration, consistent with reduced photochemical quenching and estimated electron transport under the tested conditions. Under C4 treatment, *A. corniculatum* qP and ETR decreased by 51.2% and 53.7%, respectively, the most severe among all species, but direct ATP/NADPH production and individual electron-transfer complexes were not measured.

NPQ (non-photochemical quenching): Increased significantly with an increasing heavy metal concentration, consistent with enhanced non-photochemical energy dissipation, although its biochemical basis was not directly tested. *A. marina* showed the highest NPQ increase (68.9% under C4), followed by *B. gymnorrhiza* (57.3%), indicating comparatively stronger NPQ responses in these species during short-term mixture exposure.

In contrast, soil-cultivated seedlings showed no significant changes in all chlorophyll fluorescence parameters across treatments (*p* > 0.05), consistent with the smaller changes recorded in the soil-cultivation group.

### 2.6. Transcriptomic Responses to Heavy Metal Stress

**Figure 5:**
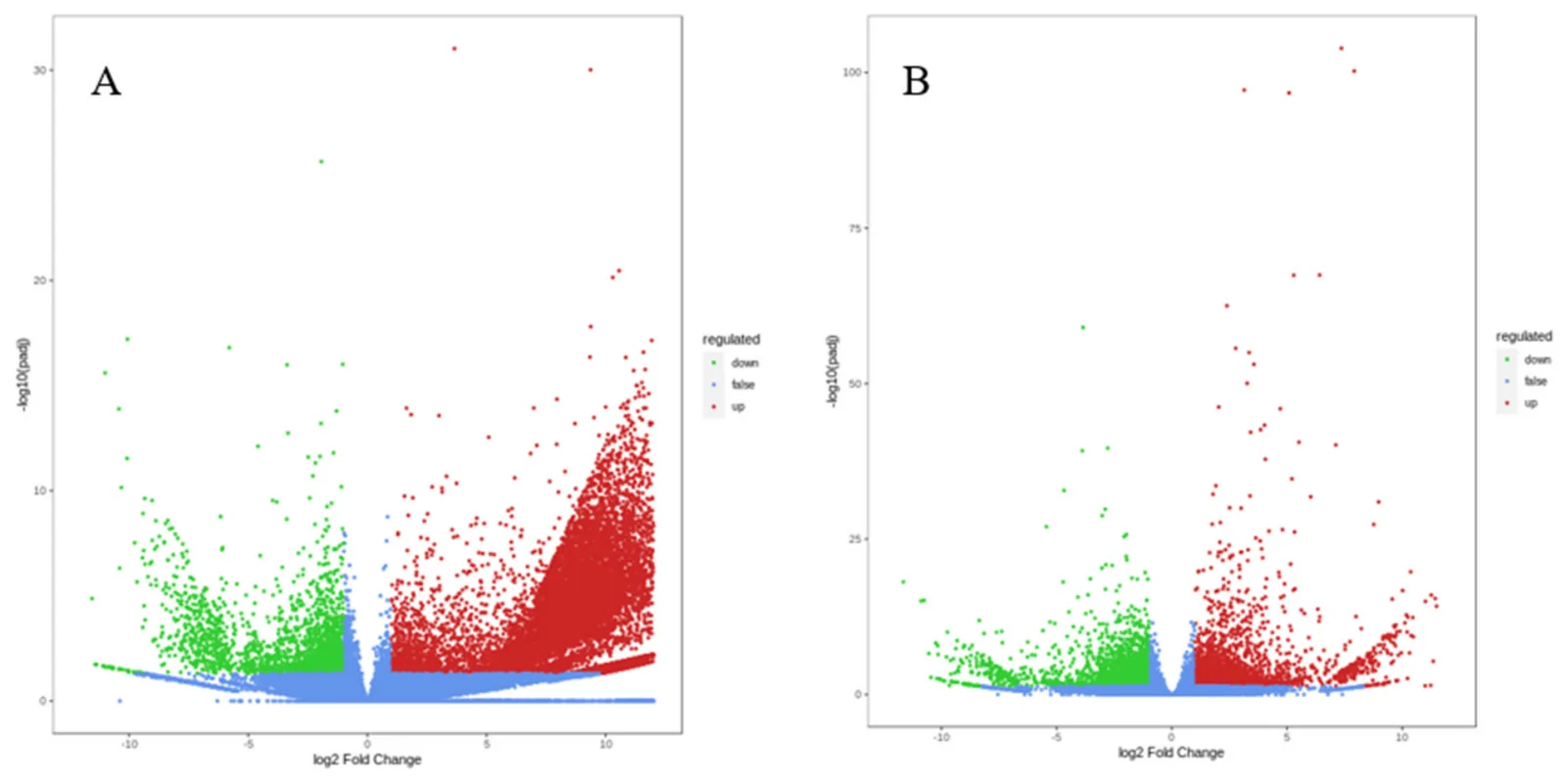
Differential gene volcano map of roots and leaves treated with high concentration of mixed heavy metals(C4). ((**A**): roots, (**B**): leaves).

**Figure 6:**
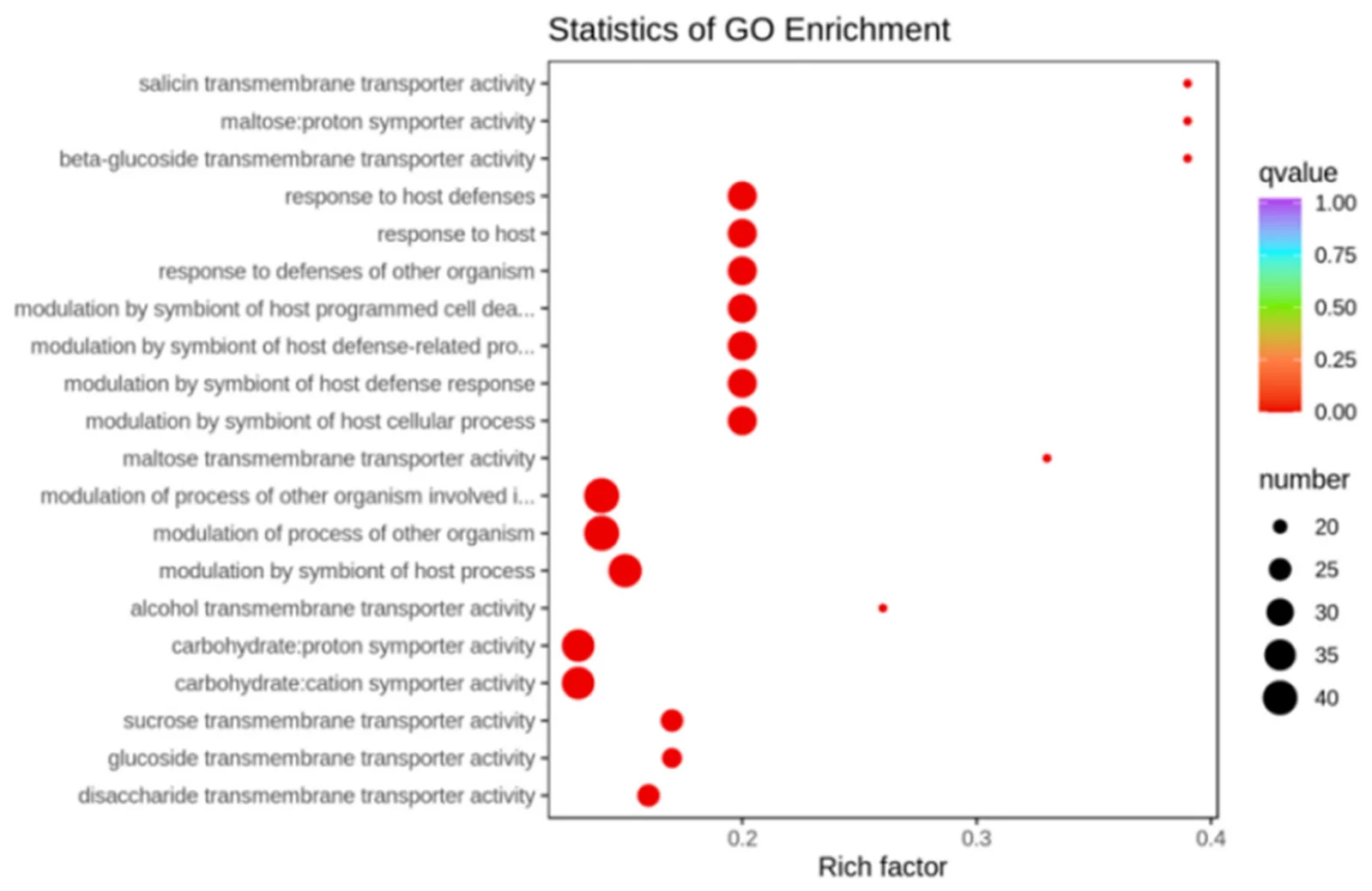
GO enrichment analysis of differential genes under high concentration of mixed heavy metals (C4) in roots.

Tables S4 and S5 show the quality test results of RNA samples and transcriptome data quality control, while Table S6 presents the functional annotation analysis. Figure S8 shows the Nr database matching the species distribution, while Figure S9 presents a bar graph of GO function classification. The reported transcriptome analysis indicated more differentially expressed genes (DEGs) in root than in leaf samples, but this tissue contrast remains exploratory until RNA-seq replication and statistical thresholds are documented (Figure 5). For the C4 mixture exposure, the reported root annotations included genes potentially associated with transport and stress responses (Figure 6). Annotated genes included putative ZIP and ABC transporters, metal-associated ATPases, metal-binding proteins, and redox-related functions; these annotations alone do not demonstrate altered transport, chelation, or enzyme activity. The reported leaf annotations included photosynthetic antenna and photosystem-related functions; experimental validation is needed before inferring a regulatory mechanism (Table S7, Figure S10).

**Figure 7:**
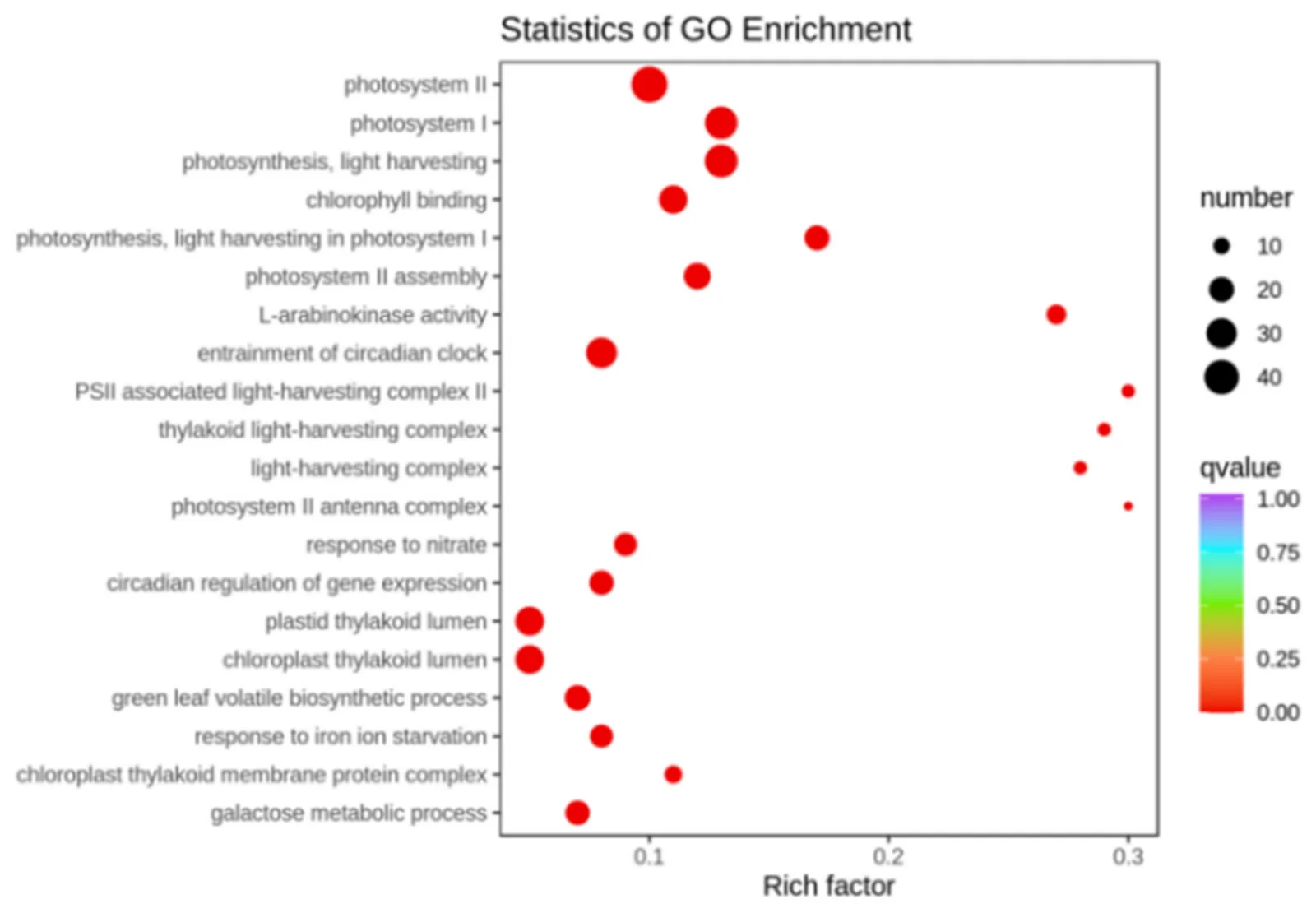
GO enrichment analysis of differential genes under high concentration of mixed heavy metals (C4) in leaves.

The original GO analysis of leaf samples reported annotations associated with PSII, PSI, light-harvesting complexes, and thylakoid components, although adjusted significance thresholds were not provided (Figure 7). Among them, the “photosynthetic antenna protein pathway” was significantly enriched in leaves, with DEGs predominantly consisting of light-harvesting complexes (LHCs). These associations do not, on their own, establish a causal link between transcript abundance and ROS homeostasis. Notably, tolerant species (*B. gymnorrhiza*, *A. marina*) were described as showing photosynthesis-related expression differences, but confirmation of between-species contrasts requires the full RNA-seq analysis details.

Plant photosynthesis can be divided into photochemical reactions and carbon reactions. In photochemical reactions, plants absorb light energy and convert it into electrical energy, which is then transformed into active chemical energy (e.g., ATP and NADPH) through photophosphorylation. Subsequently, in carbon reactions, ATP and carbon dioxide are activated by enzymes to synthesize carbohydrates [19]. It is well-established that electron transport fluxes in PSII (both donor and acceptor sides) may be disrupted by environmental factors such as temperature, drought, and heavy metals [20,21]. Studies have demonstrated that, under Pb and Cd stress, chloroplast numbers decrease or structural integrity is compromised, ultimately accelerating chlorophyll degradation [22,23].

## 3. Discussion

### 3.1. Heavy Metal Uptake and Transport in Mangrove Roots

Measured metal concentrations in this experiment were higher in mangrove roots than in leaves for Cu, Pb, and Cd, with root accumulation significantly higher than in leaves across all treatments. Possible mechanisms described in the literature include metal binding to root cell walls, vacuolar sequestration, and rhizosphere complexation; these processes were not separately measured here, so root retention cannot be attributed to a specific pathway [10].

Hydroponically grown seedlings showed higher root metal accumulation than soil-grown seedlings; this comparison demonstrates a difference in plant uptake but does not directly quantify solution- or pore-water bioavailability. Metal binding to organic matter, clay, or mineral phases is one plausible explanation for lower soil-system uptake, but metal fractionation was not measured [8]. The reported 36-fold difference in root Cu content should therefore be interpreted as an uptake contrast under two distinct cultivation systems rather than a quantitative bioavailability estimate.

Interspecific differences in heavy metal accumulation were evident, especially for Cd. *A. marina* accumulated the lowest root Cd content, alongside its comparatively small physiological changes (minimal morphological damage, stable chlorophyll content, low H_2_O_2_ accumulation). Possible explanations include transporter regulation, chelation, and subcellular sequestration, but none was directly validated in the present experiment [24]. In contrast, *K. obovata* accumulated the highest levels of Cu, Pb, and Cd in roots, contributing to its high sensitivity. These differences warrant targeted tests of uptake kinetics and metal localization among species.

### 3.2. Effects of Heavy Metals on Mangrove Growth and Photosynthesis

Heavy metal stress significantly inhibited mangrove growth, with more severe effects in hydroponic than soil systems, and was accompanied by higher measured metal accumulation in hydroponically grown plants. Reduced nutrient or water uptake and changes in photosynthesis are plausible contributors to growth inhibition, although root water uptake and gas exchange were not measured directly. Heavy metals induce root cell membrane lipid peroxidation, disrupting integrity and increasing permeability, thereby inhibiting absorption of essential nutrients (N, P, K, Mg) and water [25]. Mg deficiency directly impairs chlorophyll synthesis, while reduced photosynthetic carbon fixation limits the carbohydrate supply for growth [26]. Related studies in maize [27] and rice [28] describe stress-associated growth changes, although responses depend on species and experimental conditions.

Morphological observations further confirmed interspecific tolerance differences: *A. corniculatum* and *K. obovata* were most sensitive, showing severe leaf yellowing and abscission under high-concentration hydroponic stress; *B. gymnorrhiza* and *A. marina* maintained intact morphology. The 100% survival rate reported for soil-grown seedlings applies only to the 30-day study period and should not be extrapolated to field restoration success. Future field studies could examine whether sediment characteristics modify metal uptake, seedling performance, and longer-term survival.

Heavy metal stress induced significant H_2_O_2_ accumulation, with levels positively correlated with morphological damage severity. Sensitive species (*A. corniculatum*, *K. obovata*) showed the highest H_2_O_2_ content, consistent with a greater oxidative burden. *A. marina* and *B. gymnorrhiza* showed lower H_2_O_2_ concentrations, but SOD and CAT were not assayed in this experiment [29]. H_2_O_2_ is a stable ROS that diffuses across cell membranes, damaging lipids, proteins, and nucleic acids, exacerbating leaf senescence and wilting, which makes H_2_O_2_ a relevant indicator of oxidative stress, without specifying the biochemical cause of the observed tissue damage.

### 3.3. Disruption of Photosynthetic Electron Transport

Chlorophyll content, a key indicator of photosynthetic capacity, decreased significantly under high-concentration heavy metal stress during the fixed-ratio Cu–Pb–Cd mixture treatment; the contribution of Cd cannot be separated from the effects of Cu and Pb in this design. This is consistent with previous studies showing that Cd and Pb suppress chlorophyll synthesis enzymes (δ-aminolevulinic acid dehydratase, protochlorophyllide reductase) and damage chloroplast ultrastructure (thylakoid membrane degradation, grana destruction), inhibiting synthesis and promoting degradation [22,30]. The slight chlorophyll increase under low-concentration stress (C1) in tolerant species could reflect a non-monotonic response; protein synthesis and carotenoid-mediated effects were not evaluated [31,32]. However, high concentrations (C3–C4) override this effect, leading to significant chlorophyll loss.

Changes in fluorescence variables indicate reduced PSII photochemical performance during mixture exposure, but they do not identify the molecular site of injury. Lower F_v_/F_m_ is consistent with photoinhibition, while lower qP and estimated ETR reflect changes in PSII photochemistry; neither ATP/NADPH production nor carbon fixation was measured. The water-splitting complex, LHCII assembly, and cytochrome b_6_f function are possible sites discussed in previous studies, but no protein-level or structural evidence was collected here [17]. Increased NPQ is consistent with greater non-photochemical energy dissipation, but a causal reduction in reactive oxygen species production was not established. Tolerant species (*A. marina*, *B. gymnorrhiza*) showed higher NPQ increases, consistent with their smaller changes in chlorophyll fluorescence under these laboratory conditions.

Previous experiments with isolated photosystem I preparations have reported structural and functional responses to Pb^2+^ exposure [17]. Such observations provide hypotheses about possible photosystem I targets but cannot establish Pb^2+^ binding to plastocyanin in the five mangrove species tested here. Our fluorescence results do not distinguish a Cu-, Pb-, or Cd-specific mechanism, and no direct assessment of photosystem I binding was undertaken.

Soil-grown plants showed lower metal accumulation and comparatively small physiological changes under the tested conditions; without pore-water measurements, a change in metal bioavailability cannot be demonstrated directly. Sediment chemistry may contribute to these differences, but recommendations about sediment amendment or restoration performance require direct speciation measurements and field testing.

### 3.4. Environmental Implications for Mangrove Restoration

The comparison between hydroponic and soil-culture systems highlights the importance of metal bioavailability in interpreting plant stress responses in mangrove wetlands. Hydroponic exposure was associated with stronger measured uptake and physiological changes, while soil cultivation showed lower plant uptake and smaller changes in fluorescence variables; metal species and bioavailable fractions were not quantified. This comparison indicates the importance of evaluating sediment properties, metal speciation, and pore-water concentrations in future studies, rather than assuming reduced bioavailability from plant uptake alone. Recent reviews document the diversity of mangrove metal-retention processes and the importance of sediment chemistry in determining ecological exposure [33,34].

From a restoration perspective, species selection should consider not only survival under metal exposure but also the capacity to maintain photosynthetic function and restrict metal translocation to aboveground tissues. Within this 30-day controlled study, *B. gymnorrhiza* and *A. marina* showed comparatively lower H_2_O_2_ concentrations and smaller changes in chlorophyll and fluorescence measurements, making them preliminary candidates for field evaluation. No composite tolerance index was calculated; the descriptive species ordering is based on visible injury, seedling height, H_2_O_2_, chlorophyll, and fluorescence patterns, and its stability across environments is unknown. Long-term field trials with metal speciation, salinity variation, and survival monitoring are needed before either species can be recommended for restoration.

## 4. Materials and Methods

### 4.1. Plant Materials and Experimental Design

Seedlings of five mangrove species (*B. gymnorrhiza*, *R. stylosa*, *K. obovata*, *A. corniculatum*, *A. marina*) were collected from a commercial nursery in Dongguan, Guangdong Province, and propagated from the seeds of natural mangrove populations. Uniform-sized seedlings (height: 25–50 cm, stem diameter: 0.5–1.0 cm) with healthy growth (no pests, diseases, or physical damage; consistent leaf number and root development) were selected. Prior to formal treatment, seedlings were acclimated in a greenhouse for 2 weeks and irrigated with 1/2 Hoagland nutrient solution containing 10% NaCl to simulate mangrove habitat salinity and adapt to artificial cultivation conditions.

The experiment adopted a completely randomized design with two cultivation methods (hydroponic culture, H; soil culture, S) and five exposure levels (an unamended control and four fixed-ratio Cu–Pb–Cd mixture treatments; Table 1). Published sediment concentrations from the Pearl River Estuary were used as contextual background when selecting the laboratory gradient, but sediment total concentrations (mg/kg) are not directly comparable with the nominal concentrations of added metal ions (mg/L). Each species-by-cultivation-by-exposure combination was described as having three biological replicate groups, each containing three seedlings; the treatment group represents the reported level of biological replication; the statistical role of individual seedlings cannot be determined from the summary dataset. Seedlings were cultivated in an artificial incubator with a 12 h light (light intensity of 20,000 lux, 25 °C)/12 h dark (25 °C) cycle.

The Cu, Pb, and Cd doses in Table 1 are nominal concentrations of added metals in the nutrient solution (mg/L); C1–C4 increase the three metals together in a fixed 25:5:1 mass ratio, using intermediate doses at C2 and C3 to describe responses along the chosen gradient. Values reported for mangrove sediments (mg/kg) were used only to place the treatments in a regional contamination context, not to convert the sediment total metal content into dissolved exposure. The dissolved, complexed, precipitated, and sorbed fractions depend on pH, salinity, ligand availability, and sediment characteristics; these fractions were not quantified. Thus, the hydroponic treatments represent controlled mixture-exposure scenarios rather than measured in situ pore-water concentrations, and this fixed-ratio design does not cover other environmentally possible cross-combinations of Cu, Pb, and Cd. Soil-culture comparisons establish differences in plant uptake and physiological response under the tested conditions, but they do not directly quantify changes in bioavailability.

Soil cultivation (S): Mangrove seedlings were planted in pots filled with coastal sandy soil (pH 6.5–7.0, organic matter content 1.2%) and irrigated every 2–3 days with four levels of mixed-metal 1/2 Hoagland nutrient solution (10% NaCl). The soil was collected from an unpolluted mangrove area in Zhanjiang, Guangdong, and provided a soil-based comparison under controlled cultivation, without direct measurement of metal speciation in soil or pore water.

Hydroponic culture (H): Seedlings were fixed on foam boards and cultured in 1/2 Hoagland nutrient solution (10% NaCl) containing the same four heavy metal gradients. The nutrient solution was replaced every 2–3 days; concentrations between replacements and the dissolved speciation of each metal were not independently verified.

After 30 days of treatment, seedling height and visible morphological changes were recorded and photographed; this exposure duration does not establish long-term tolerance or field survival. Root and leaf samples were collected to determine heavy metal content, chlorophyll content, chlorophyll fluorescence parameters, and H_2_O_2_ content [35,36,37].

### 4.2. Determination of Cu, Pb, and Cd Contents

Root and leaf samples (1 g fresh weight, FW) were rinsed with deionized water, heated at 105 °C for 30 min and dried to constant weight at 80 °C (approximately 8 h). Dried samples were ground into powder and passed through a 100-mesh sieve. Exactly 0.1 g of sieved powder was weighed into a polytetrafluoroethylene digestion tank, mixed with 4 mL concentrated nitric acid (65%, analytical grade), and digested using a microwave digestion system (CEM MARS 6, Matthews, NC, USA) under optimized conditions: 5 min heating to 120 °C (holding 5 min), 5 min heating to 180 °C (holding 15 min). After cooling, the digest was transferred to a 25 mL volumetric flask, diluted with deionized water, and mixed thoroughly. Cu, Pb, and Cd contents were determined by inductively coupled plasma–mass spectrometry (ICP-MS, Agilent 790, Santa Clara, CA, USA).

The metal concentration was reported in μg/g dry weight (DW) after accounting for digest volume and dry sample mass. When ICP–MS output is expressed in mg/L, the dimensional conversion is C (mg/L) × final digest volume (mL)/sample dry mass (g), yielding μg/g; any additional dilution must be included separately.

Blank controls (digestion without samples) and standard reference materials (GBW07603 Poplar Leaves) were included to ensure accuracy, with a recovery rate of 95–105%. Each sample was analyzed in three technical replicates.

### 4.3. Chlorophyll Content Measurement

Chlorophyll content was determined by ethanol extraction spectrophotometry. Briefly, 0.2 g of fresh leaf tissue (avoiding main veins) was cut into 1 mm × 1 mm pieces and placed in a 10 mL centrifuge tube with 4 mL 95% ethanol (analytical grade). The sealed tube was kept at 4 °C in darkness for 72 h and shaken gently every 24 h. After extraction, the supernatant was obtained by centrifugation at 5 × 10^3^× *g* for 5 min. Absorbance values were measured at 470 nm (carotenoids), 649 nm (chlorophyll b), and 665 nm (chlorophyll a) using a spectrophotometer (Shimadzu UV-2600, Kyoto, Japan), with 95% ethanol as the blank control. Total chlorophyll content (mg/g FW) was calculated as:

Chlorophyll a (Chl a) = 13.9 A_665_ − 6.8 A_649_

Chlorophyll b (Chl b) = 24.9 A_649_ − 7.3 A_665_

Total chlorophyll = Chl a + Chl b

### 4.4. Chlorophyll Fluorescence Parameters Measurement

Chlorophyll fluorescence parameters were measured using a portable chlorophyll fluorescence spectrometer (PAM-2500, Walz, Effeltrich, Germany), a non-destructive method to evaluate photosynthetic apparatus function (especially PSII). Prior to measurement, seedlings were dark-adapted for 30 min to fully oxidize the PSII reaction center (all centers open, a prerequisite for accurate parameter determination). After dark adaptation, minimum fluorescence (F_o_, open reaction centers) and maximum fluorescence (F_m_, closed reaction centers) were measured under saturation pulse light (6000 μmol m^−2^ s^−1^, duration 0.8 s). Maximum quantum yield of PSII (F_v_/F_m_ = (F_m_ − F_o_)/F_m_) was calculated.

Subsequently, actinic light (300 μmol m^−2^ s^−1^, simulating natural growth light intensity) was activated. After fluorescence stabilization (≈5 min), steady-state fluorescence (F_s_) was measured. A saturation pulse was reapplied to determine maximum fluorescence under light-adapted conditions (F_m_′). Photochemical quenching coefficient (qP = (F_m_′ − F_s_)/(F_m_′ − F_o_′)), non-photochemical quenching (NPQ = (F_m_ − F_m_′)/F_m_′), and electron transport rate (ETR = 0.5 × 0.84 × PAR × ΦPSII, where ΦPSII = (F_m_′ − F_s_)/F_m_′) were considered in the interpretation of the instrument outputs (0.5 = assumed energy partitioning to PSII; 0.84 = assumed leaf absorptance). Each measurement was repeated three times on different leaves of the same seedling, with the average value used as the final result.

### 4.5. Leaf H_2_O_2_ Assay

Leaf H_2_O_2_ content was determined using a commercial assay kit (Nanjing Jiancheng Bioengineering Institute, Nanjing, China) based on the yellow complex formed by H_2_O_2_ and molybdic acid, with absorbance at 405 nm positively correlated with H_2_O_2_ content [31]. Fresh leaf tissue (0.5 g) was ground into a homogenate with 4 mL ice-cold phosphate buffer (50 mmol/L, pH 7.8; ice grinding prevents H_2_O_2_ decomposition and enzyme inactivation). The homogenate was centrifuged at 12,000× *g* for 20 min at 4 °C to remove cell debris. Supernatant (0.1 mL) was mixed with 0.9 mL assay reagent and incubated at 37 °C for 30 min, and absorbance was measured at 405 nm. H_2_O_2_ content (μmol/g FW) was calculated based on a standard curve.

### 4.6. RNA Sequencing and Transcriptome Analysis

RNA was extracted from plant tissues using TRIzol reagent (Invitrogen, Waltham, MA, USA), and its purity was evaluated using 1% agarose gel electrophoresis and a NanoDrop 2000 spectrophotometer (Thermo Fisher Scientific, Waltham, MA, USA). Root and leaf tissue were analyzed by transcriptome sequencing after library quality assessment using an Illumina HiSeq platform (Illumina, San Diego, CA, USA). The documented sequencing description does not specify the number of independent RNA-seq libraries per group, sequencing depth, read trimming and quality-control parameters, reference genome or de novo assembly method, mapping software, differential-expression thresholds, multiple-testing correction, or the GO annotation/enrichment workflow. Accordingly, the gene-level and GO-enrichment patterns are considered exploratory and cannot presently be reproduced independently from the reported methods.

### 4.7. Data Analysis

All data were processed with Microsoft Excel 2019 and expressed as the mean ± standard deviation (SD) of three biological replicates. Statistical analysis was performed using GraphPad Prism 6.0 software. One-way analysis of variance (ANOVA) followed by Dunnett’s multiple comparison test was used for normally distributed data (Shapiro–Wilk normality test); Dunn’s multiple comparison test was used for non-parametric data. Pearson correlation analysis was performed to analyze correlations between heavy metal accumulation, H_2_O_2_ content, gene expression, and photosynthetic parameters. Differences were considered statistically significant at *p* < 0.05. Figures were produced using the online platform at https://www.bioinformatics.com.cn (accessed on 30 June 2026).

## 5. Conclusions

The five mangrove species differed in growth, visible injury, tissue metal accumulation, H_2_O_2_ content, and photosynthetic fluorescence during 30 days of controlled Cu–Pb–Cd mixture exposure. Roots contained more of the measured metals than leaves, and soil-grown seedlings showed lower tissue metal concentrations than hydroponically grown seedlings. Because dissolved and pore-water metal fractions were not measured, this uptake difference cannot be taken as direct proof of reduced metal bioavailability in soil. The absence of single-metal controls also precludes conclusions about additive, synergistic, or antagonistic interactions.

Higher mixture concentrations were associated with lower chlorophyll content and PSII-related photochemical parameters and with increased H_2_O_2_ content, particularly in *A. corniculatum* and *K. obovata*. *B. gymnorrhiza* and *A. marina* showed comparatively smaller short-term physiological changes. The descriptive pattern *B. gymnorrhiza* > *A. marina* > *R. stylosa* > *K. obovata* > *A. corniculatum* summarizes observations under the tested conditions and is not a validated composite tolerance ranking. Transcriptomic associations suggest targets for follow-up experiments but do not demonstrate the underlying protein-level mechanisms. Antioxidant-enzyme activities and field performance were not established in the present work; consequently, *B. gymnorrhiza* and *A. marina* should be regarded as preliminary candidates for long-term and field evaluation.

## Figures and Tables

**Table 1:** Nominal added concentrations of Cu, Pb, and Cd in the mixture treatments (mg/L).

Mixed Heavy Metal (mg/L)	Cu^2+^	Pb^2+^	Cd^2+^
Control(CK)	0	0	0
C1	5	1	0.2
C2	25	5	1
C3	50	10	2
C4	75	15	3

## Data Availability

The data presented in this study are available on request from the corresponding author. The data are not publicly available due to privacy reason.

## Supplementary Materials

The following supporting information can be downloaded at https://www.mdpi.com/article/10.3390/plants15203197/s1: Figure S1: The Cu contents in the leaves and roots of five mangroves; Figure S2: The Pb contents in the leaves and roots of five mangroves; Figure S3: The Cd contents in the leaves and roots of five mangroves; Figure S4: Effect of heavy metal stress on the phenotypes of Five species of mangroves plants (A: *Bruguiera gymnorrhiza*, B: *Rhizophora stylosa*, C: *Kandelia obovata*, D: *Aegiceras corniculatum*, E: *Avicennia marina*); Figure S5: The H_2_O_2_ content of five mangroves with Hydroponic culture treatment. Values and error bars indicate means ± standard deviation (*n* = 3). The different letters in the same column mean a significant difference (*p* < 0.05); Figure S6: Chlorophyll concentration of five mangrove species under hydroponic combined-metal exposure. Values and error bars indicate mean ± standard deviation (*n* = 3). Different letters indicate significant differences among treatments within the same species (*p* < 0.05); Figure S7: Effects of combined Cu–Pb–Cd stress on chlorophyll fluorescence parameters of five mangrove species under hydroponic exposure: F_v_/F_m_ (A), ETR (B), qP (C), and NPQ (D). Values and error bars indicate mean ± standard deviation (*n* = 3); Figure S8: Nr database matching species distribution; Figure S9: Bar graph of GO function classification; Figure S10: KOG functional annotation distribution of unigenes in transcriptome for mangrove plants; Table S1: Statistical comparison of Cu, Pb, and Cd accumulation between roots and leaves and between cultivation systems; Table S2: Dunn’s multiple comparison test for root Cd accumulation among five mangrove species under hydroponic exposure; Table S3: Effects of combined Cu–Pb–Cd treatments on seedling height of five mangrove species; Table S4: Quality test results of RNA samples; Table S5: Quality control of transcriptome results; Table S6: Functional annotation analysis; Table S7: KOG classification.
