# Supplementary material for: Ecophysiological and Transcriptomic Responses of Mangrove Seedlings to Environmentally Relevant Cu–Pb–Cd Combined-Metal Stress
Source: Plants (Basel). 2026 Oct 18;15(20):3197. doi: 10.3390/plants15203197 (PMC13709257; doi:10.3390/plants15203197)
Supplement: Supplementary file 1 [file plants-15-03197-s001.zip › plants-4582748-supplementary.pdf]

Supplementary Material

Table S1. Statistical comparison of Cu, Pb, and Cd accumulation between roots and leaves and between cultivation systems

	P value	Cu concentrations	Pb concentrations	Cd concentrations
Soil culture method	root Vs leaf	$P < 1 \times 10^{-4}$***	$P < 1 \times 10^{-4}$***	$P < 1 \times 10^{-4}$***
Hydroponic method	root Vs leaf	$P < 1 \times 10^{-4}$***	$P < 1 \times 10^{-4}$***	$P < 1 \times 10^{-4}$***
root	Soil culture method vs Hydroponic method	$P < 1 \times 10^{-4}$***	1.46×10^{-2}	1.5×10^{-3}**
leaf	Soil culture method vs Hydroponic method	$P < 1 \times 10^{-4}$***	$P < 1 \times 10^{-4}$***	0.996

Note: *** indicates a significant correlation at $P < 1 \times 10^{-4}$. * *indicates a significant correlation at $P < 0.01$.

Table S2. Dunn’s multiple comparison test for root Cd accumulation among five mangrove species under hydroponic exposure

Dunn's Multiple Comparison Test	Difference in rank sum	Significant? $P < 0.05$?	Summary
B.g vs *R.s*	-2.50	No	ns
B.g vs *K.o*	-1.00	No	ns
B.g vs *A.c*	-32.0	No	ns
B.g vs *A.m*	-69.5	Yes	***
R.s vs *K.o*	1.50	No	ns
R.s vs *A.c*	-29.5	No	ns
R.s vs *A.m*	-67.0	Yes	**
K.o vs *A.c*	-31.0	No	ns
K.o vs *A.m*	-68.5	Yes	***
A.c vs *A.m*	-37.5	No	ns

Note: *** indicates a significant correlation at $P < 0.001$. ** indicates a significant correlation at $P < 0.05$.

Table S3. Effects of combined Cu–Pb–Cd treatments on seedling height of five mangrove species

	Mixed with heavy metal treatment	*B.gymnorrhiza*	*R.stylosa*	*K.obovata*	*A.corniculatum*	*A.marina*
Soil culture method	CK	33.3±0.12a	50.4±0.34a	30.9±0.53a	27.6±0.46a	26.3±0.36a
	C1	34.3±0.33a	51.3±0.45a	32.7±0.76a	26.6±0.21a	27.8±0.48a
	C2	33.4±0.24a	49.4±0.26a	29.3±0.56a	24.5±0.43a	25.7±0.58a
	C3	32.2±0.14a	48.2±0.43a	28.1±0.16a	23.4±0.25a	24.5±0.34a
	C4	30.1±0.21a	47.2±0.41a	26.1±0.10a	22.3±0.23a	23.1±0.22a
Hydroponic method	CK	34.7±0.24a	52.2±0.32a	32.7±0.10a	25.3±0.21a	27.7±0.16a
	C1	35.6±0.26a	53.2±0.41a	32.9±0.10a	25.9±0.32a	27.9±0.14a
	C2	31.4±0.21b	51.2±0.23b	30.6±0.10b	23.3±0.24b	23.8±0.23b
	C3	30.6±0.35b	49.2±0.12b	25.8±0.10c	17.3±0.15c	20.6±0.21b
	C4	28.1±0.23c	45.2±0.24c	20.9±0.10d	10.3±0.20d	19.05±0.16c

Note: The different letters in the same column mean a significant difference ($P<0.05$)

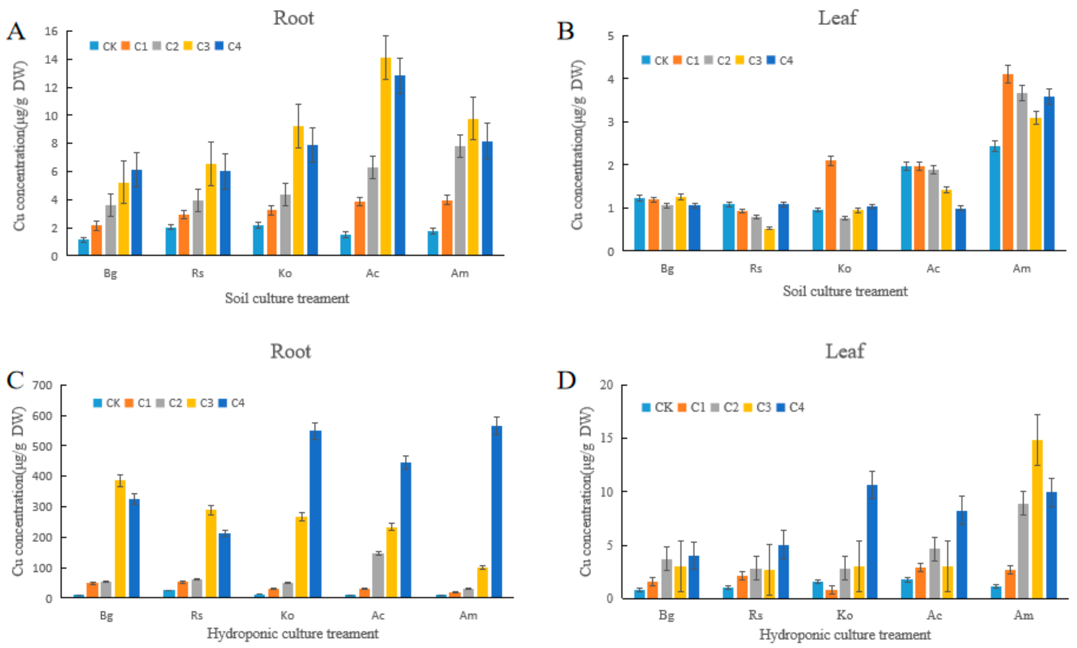

Figure S1. The Cu contents in the leaves and roots of five mangroves

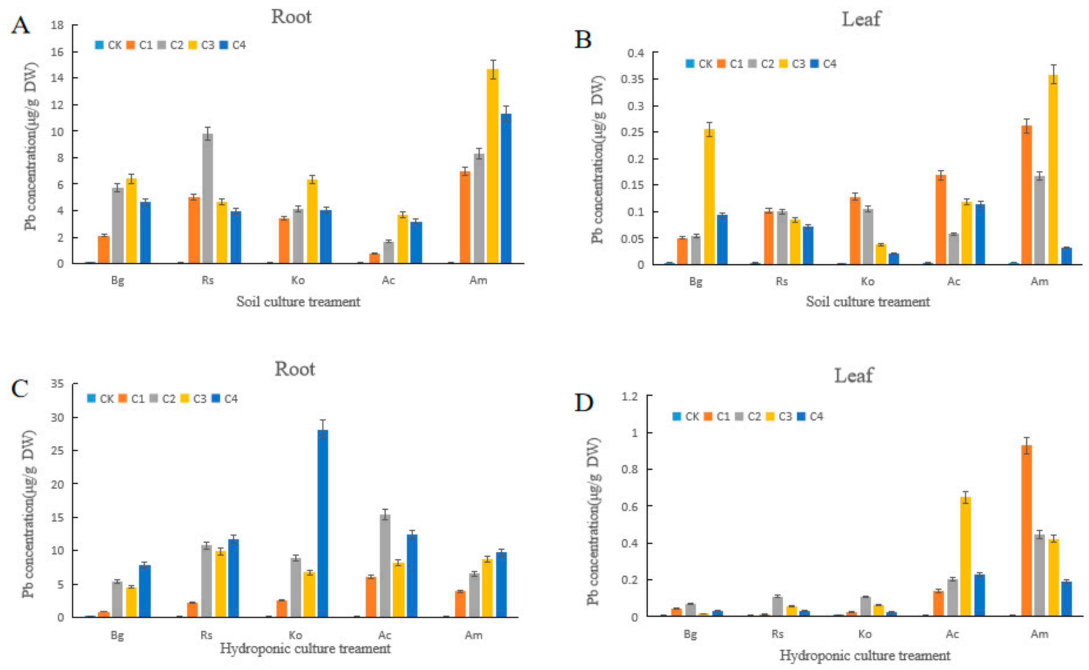

Figure S2. The Pb contents in the leaves and roots of five mangroves

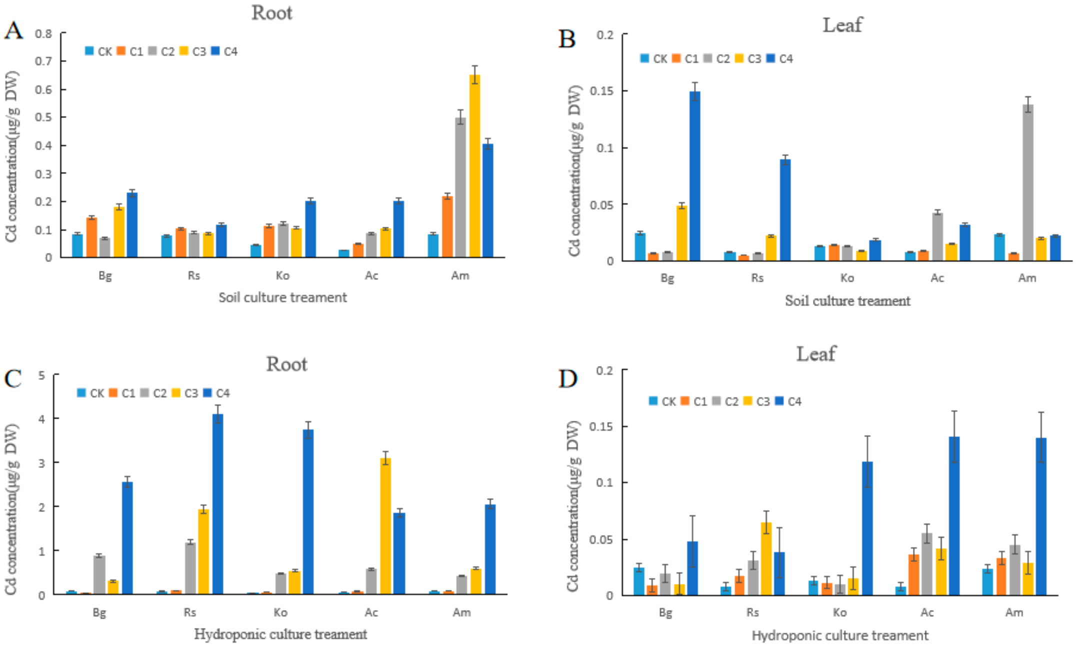

Figure S3. The Cd contents in the leaves and roots of five mangroves

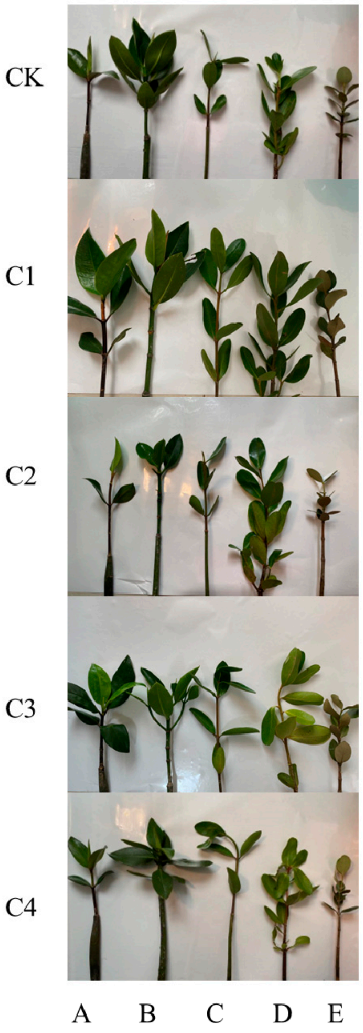

Figure S4. Effect of heavy metal stress on the phenotypes of Five species of mangroves

plants(A: *Bruguiera gymnorrhiza,* B: *Rhizophora stylosa*, C: *Kandelia obovata*, D: *Aegiceras corniculatum*, E: *Avicennia marina*)

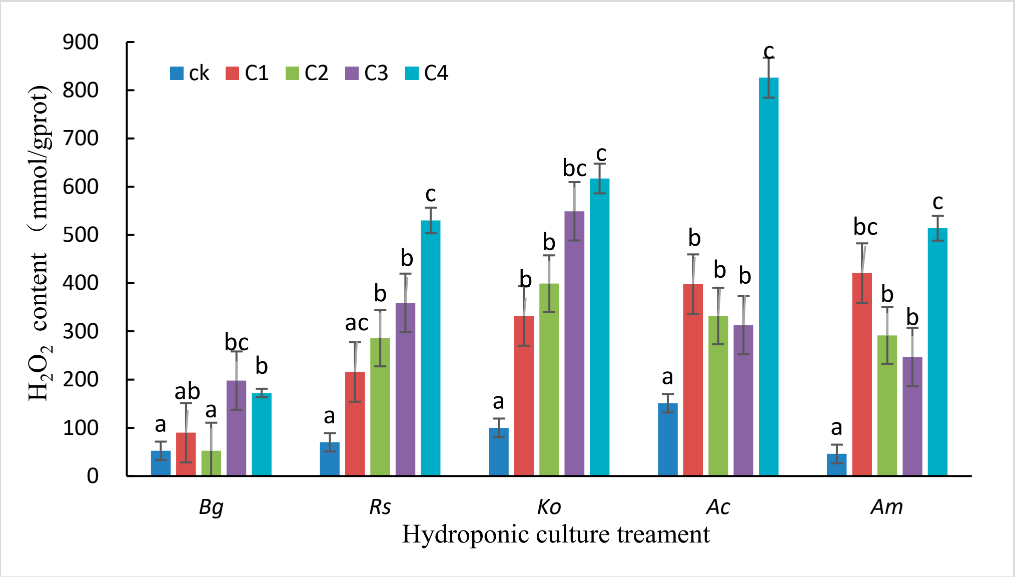

Figure S5. The H_2O_2 content of five mangroves with Hydroponic culture treatment.Values and error bars indicate means ± standard deviation (n = 3). The different letters in the same column mean a significant difference (*P*<0.05)

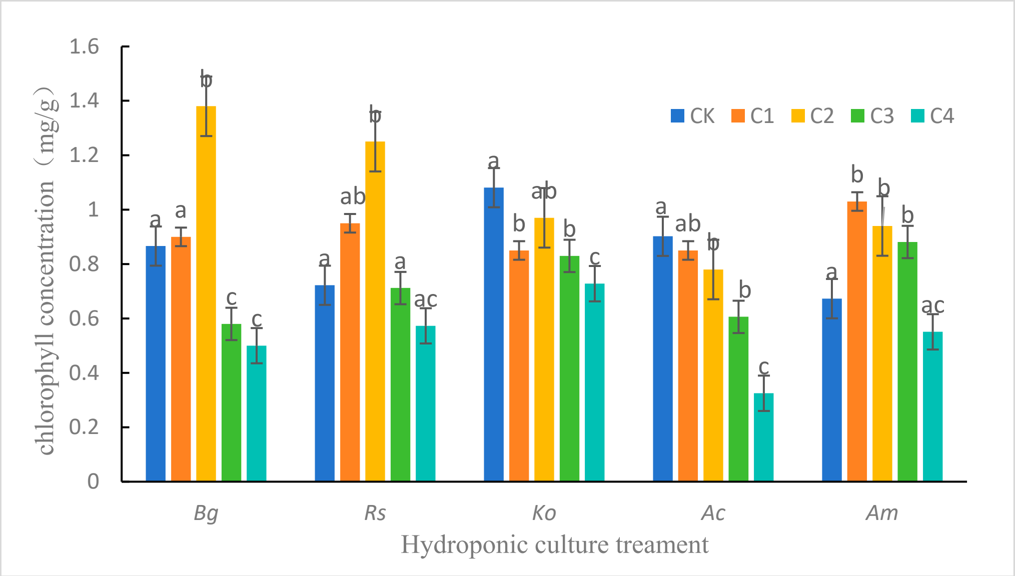

Figure S6. Chlorophyll concentration of five mangrove species under hydroponic combined-metal exposure. Values and error bars indicate mean ± standard deviation (n = 3). Different letters indicate significant differences among treatments within the same species (*P* < 0.05).

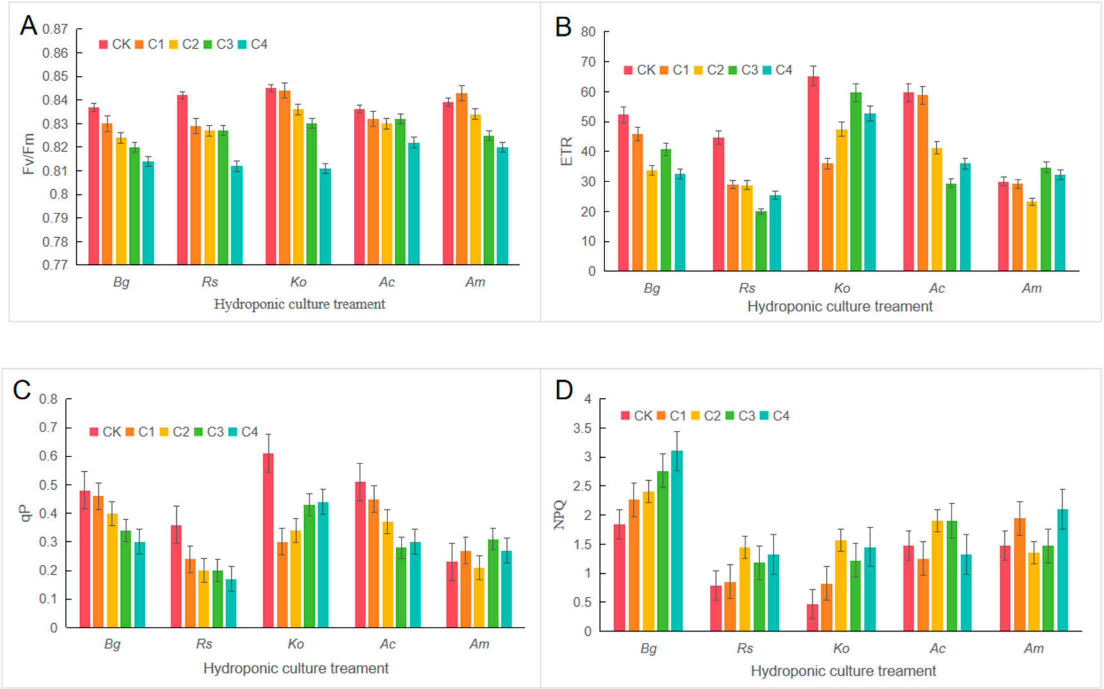

Figure S7. Effects of combined Cu–Pb–Cd stress on chlorophyll fluorescence parameters of five mangrove species under hydroponic exposure: F_v/F_m (A), ETR (B), qP (C), and NPQ (D). Values and error bars indicate mean ± standard deviation (n = 3).

Table S4. Quality test results of RNA samples

No.	Sample Name	Concentration (ng/ μL)	Volume(μL)	Total Amount(u g)	Integrity Value	Test Result
1	BgCK-S1	147	35	5.145	9.6	Pass
2	BgCK-S2	67	35	2.345	8.5	Pass
3	BgCK-S3	89	35	3.115	7.8	Pass
4	BgC4-S1	79	35	2.765	7.3	Pass
5	BgC4-S2	129	35	4.515	7.3	Pass
6	BgC4-S3	30	35	1.05	8.8	Pass
7	BgC1-S1	65	35	2.275	8.6	Pass

8	BgC1-S2	118	35	4.13	8.5	Pass
9	BgC1-S3	119	35	4.165	8.4	Pass
10	BgCK-L1	127	35	4.445	10	Pass
11	BgCK-L2	34	35	1.19	8.9	Pass
12	BgCK-L3	39	35	1.365	9.4	Pass
13	BgC4-L1	304	35	10.64	9.9	Pass
14	BgC4-L2	141	35	4.935	9.1	Pass
15	BgC4-L3	161	35	5.635	9.1	Pass
16	BgC1-L1	46	35	1.61	7.2	Pass
17	BgC1-L2	71	35	2.485	8.8	Pass
18	BgC1-L3	65	35	2.275	8.6	Pass
19	AcCK-S1	381	35	13.335	8.9	Pass
20	AcCK-S2	113	35	3.955	7.7	Pass
21	AcCK-S3	120	35	4.2	8.7	Pass
22	AcC4-S1	112	35	3.92	9	Pass
23	AcC4-S2	242	35	8.47	8.7	Pass
24	AcC4-S3	133	35	4.655	8.6	Pass
25	AcC1-S1	108	35	3.78	7.3	Pass
26	AcC1-S2	71	35	2.485	8	Pass
27	AcC1-S3	43	35	1.505	8.5	Pass
28	AcCK-L1	132	35	4.62	9	Pass
29	AcCK-L2	126	35	4.41	9	Pass
30	AcCK-L3	92	35	3.22	8.7	Pass

31	AcC4-L1	154	35	5.39	9.1	Pass
32	AcC4-L2	57	35	1.995	9.5	Pass
33	AcC4-L3	362	35	12.67	9.9	Pass
34	AcC1-L1	196	35	6.86	9.5	Pass
35	AcC1-L2	519	35	18.165	9.8	Pass
36	AcC1-L3	305	35	10.675	9.7	Pass

Table S5. Quality control of transcriptome results

Sample	Clean Base(G)	Error Rate (%)	Q20(%)	Q30(%)	GC Content(%)
AcCK-L1	6.9	0.03	97.27	92.59	45.5
AcCK-L2	7.18	0.03	97.12	92.26	45.5
AcCK-L3	7.07	0.03	97.15	92.31	45.62
AcCK-S1	7.05	0.03	97.35	92.77	45.29
AcCK-S2	7.22	0.03	97.2	92.48	46.62
AcCK-S3	6.82	0.03	97.26	92.61	46.62
AcC4-L1	6.9	0.03	97.52	93.12	45.4
AcC4-L2	7	0.03	97.51	93.09	45.62
AcC4-L3	7.18	0.03	97.36	92.81	45.52
AcC4-S1	6.89	0.03	97.23	92.51	45.34
AcC4-S2	6.52	0.03	97.19	92.41	45.14
AcC4-S3	7.08	0.03	97.3	92.66	47.52
AcC1-L1	6.92	0.03	97.55	93.19	45.23
AcC1-L2	7.37	0.03	97.19	92.41	45.57

AcC1-L3	7.52	0.03	97.12	92.26	45.37
AcC1-S1	7.12	0.03	97.28	92.67	46.25
AcC1-S2	7.71	0.03	97.24	92.52	45.02
AcC1-S3	6.86	0.03	97.2	92.49	46.17
BgCK-L1	7.07	0.03	97.45	92.97	45.74
BgCK-L2	8.08	0.03	97.3	92.66	45.79
BgCK-L3	6.49	0.03	97.47	93.05	45.79
BgCK-S1	6.71	0.03	97.18	92.49	46.23
BgCK-S2	7.02	0.03	97.18	92.45	46.74
BgCK-S3	6.74	0.03	97.3	92.71	47.11
BgC4-L1	7.05	0.03	97.29	92.66	45.92
BgC4-L2	7.03	0.03	97.57	93.21	46.02
BgC4-L3	6.93	0.03	97.38	92.86	46.03
BgC4-S1	6.6	0.03	97.25	92.57	46.99
BgC4-S2	7.14	0.03	97.34	92.82	48.48
BgC4-S3	6.52	0.03	97.3	92.75	50.24
BgC1-L1	7.15	0.03	97.35	92.8	45.75
BgC1-L2	6.59	0.03	97.21	92.49	45.93
BgC1-L3	6.98	0.03	97.54	93.17	45.94
BgC1-S1	7	0.03	97.35	92.79	47.56
BgC1-S2	6.87	0.03	97.38	92.9	47.33
BgC1-S3	6.99	0.03	97.37	92.87	47.34

Table S6. Functional annotation analysis

data base	quantity	percentage (%)
KEGG	197158	42.02
NR	281772	60.05
SwissProt	218091	46.48
TrEMBL	310328	66.13
KOG	215363	45.9
GO	261821	55.8
Pfam	253826	54.09
Annotated in at least one Database	346229	73.78
totality	469251	100

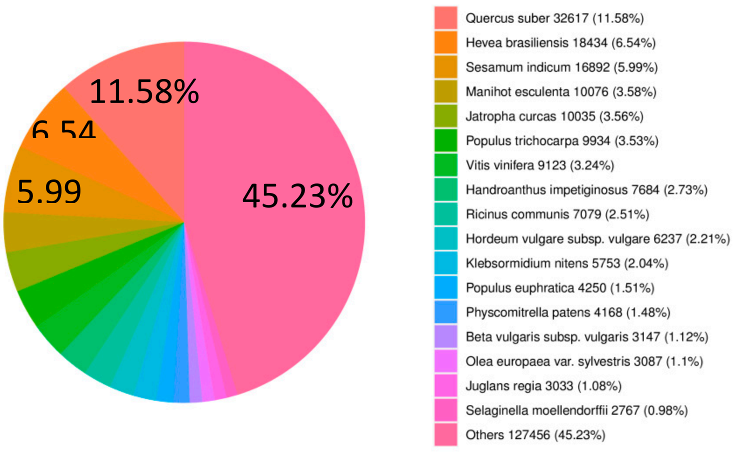

Figure S8. Nr database matching species distribution

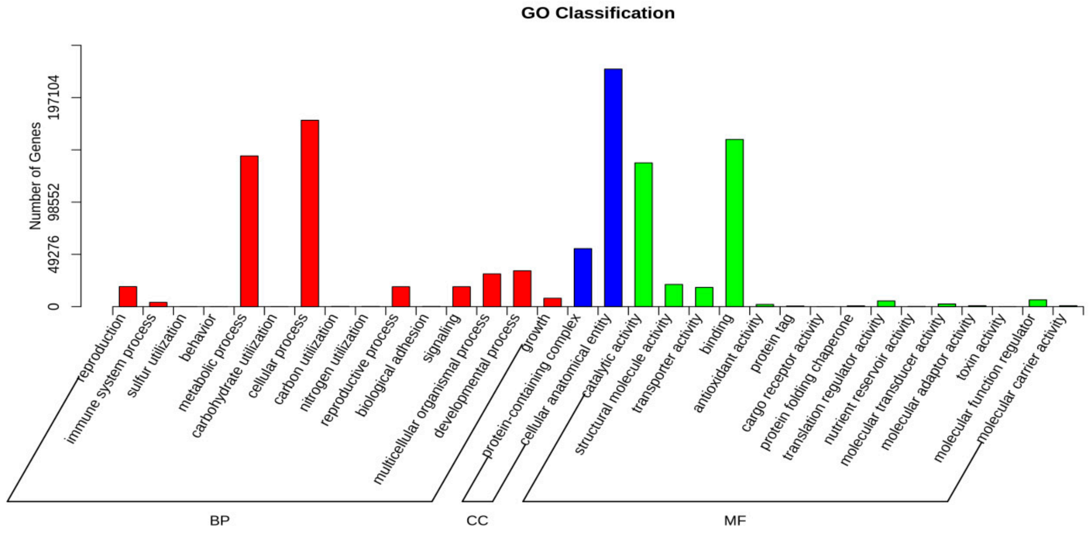

Figure S9. Bar graph of GO function classification

Table S7. KOG classification

classify	function	gene dosage	percentage (%)
R	General function prediction only	31631	13.23%
O	Posttranslational modification, protein turnover, chaperones	28733	12.02%
J	Translation, ribosomal structure and biogenesis	27646	11.56%
T	Signal transduction mechanisms	20511	8.58%
C	Energy production and conversion	13206	5.52%
U	Intracellular trafficking, secretion, and vesicular transport	11958	5.00%
G	Carbohydrate transport and metabolism	11319	4.73%
E	Amino acid transport and metabolism	10063	4.21%

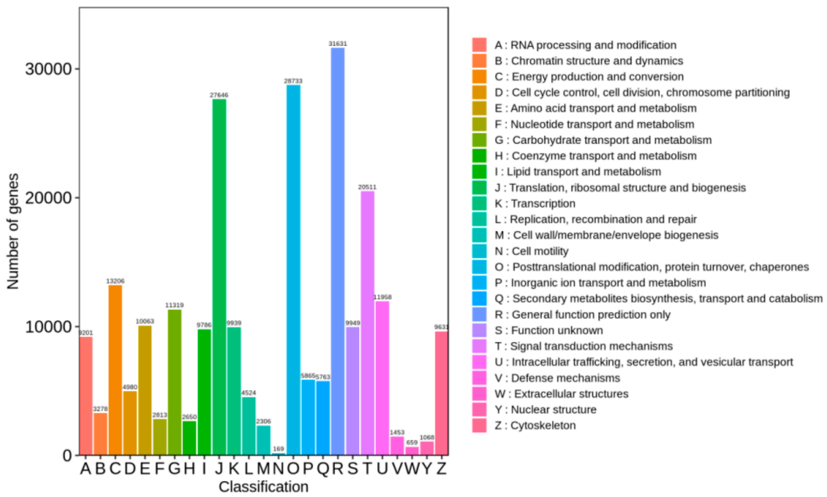

Figure S10. KOG functional annotation distribution of unigenes in transcriptome for mangrove plants
